# The Hypothalamic Nuclei Implicated in the Regulation of Polycystic Ovary Syndrome: A Review of Its Clinical, Metabolic, and Endocrine Aspects

**DOI:** 10.3390/molecules30163407

**Published:** 2025-08-18

**Authors:** Elizabeth Vieyra, Carlos-Camilo Silva, Rosa Linares, Gabriela Rosas, Julieta-Azucena Espinoza, Andrea Chaparro, Roberto Calderón, Belinda de la Peña, Leticia Morales-Ledesma

**Affiliations:** 1 Neuroendocrinology of Reproduction Research Laboratory-UIBR, Facultad de Estudios Superiores Zaragoza, Universidad Nacional Autónoma de México, Mexico City 09230, Mexico; roberto.calderon23@comunidad.unam.mx; 2 Reproductive Physiology Laboratory-UIBR, Facultad de Estudios Superiores Zaragoza, Universidad Nacional Autónoma de México, Mexico City 09230, Mexico; gabriela_rosasg@comunidad.unam.mx (G.R.); azuespinozamoreno@unam.edu (J.-A.E.); chaparrortegandrea@comunidad.unam.mx (A.C.); 3 Nursing Career, Facultad de Estudios Superiores Zaragoza, Universidad Nacional Autónoma de México, Mexico City 09230, Mexico; enf.jefe@zaragoza.unam.mx; 4 Chronobiology of Reproduction Research Laboratory-UIBR, Facultad de Estudios Superiores Zaragoza, Universidad Nacional Autónoma de México, Mexico City 09230, Mexico; centrofalia@comunidad.unam.mx; 5 Endocrinology Laboratory-UIBR, Facultad de Estudios Superiores Zaragoza, Universidad Nacional Autónoma de México, Mexico City 09230, Mexico; rosalinares@comunidad.unam.mx

**Keywords:** arcuate nucleus, gamma aminobutyric acid, hyperandrogenism, kisspeptin, neuropeptide Y, polycystic ovarian syndrome, rostral periventricular area of the third ventricle

## Abstract

Polycystic ovary syndrome (PCOS) is an endocrine and metabolic disorder characterized by a clinical and/or biochemical hyperandrogenism. In addition, PCOS is also associated with the presence of ovarian cysts, anovulation, and menstrual abnormalities such as oligomenorrhea or amenorrhea. The aetiology of the syndrome is multifactorial and heterogeneous due to the interaction of genetic, hormonal, metabolic, and environmental factors, as well as the different phenotypes and responses to treatments exhibited by the patients. Considering this complex interaction, it is essential to continue with the research focused on the mechanisms involved in the development and maintenance of the pathology. The alteration in the pulsatile secretion of the gonadotropin-releasing hormone (GnRH) is considered to be one of the main causes that contributes to its onset. In this review, we discuss recent evidence about the role of the rostral periventricular area of the third ventricle (RP3V), the arcuate nucleus (ARC), and the ventromedial nucleus of the hypothalamus (VMH), key hypothalamic regions that regulate GnRH secretion, in the development of PCOS. In addition, we analyse the clinical, metabolic, and endocrine factors that interact in the patients with PCOS, offering a multifactorial perspective to improve our understanding of this disorder.

## 1. Introduction

Polycystic ovary syndrome (PCOS) has been studied since the 1930s, and to date, no clear explanation for its aetiology has been found. In 1935, Stein and Leventhal first reported their clinical findings in seven women who presented with obesity, hirsutism, abnormalities in their menstrual cycles, enlarged ovaries with multiple cysts, and infertility. By 1990, the National Institutes of Health (NIH) in the United States proposed diagnostic criteria for PCOS based on the presence of hyperandrogenism associated with chronic anovulation, without considering morphological changes in the ovaries. In 2003, a new diagnostic consensus was established during a meeting convened in Rotterdam by the European Society of Human Reproduction and Embryology and the American Society for Reproductive Medicine [1,2]. In 2023, the new International Evidence-Based Guidelines for the Evaluation and Treatment of Polycystic Ovary Syndrome were established. The authors mention that PCOS should be diagnosed using the 2018 International Evidence-Based Guideline criteria, which is based on the consensus-based 2003 Rotterdam criteria. The current standard requires the presence of at least two of the following characteristics: (1) clinical/biochemical hyperandrogenism, (2) ovulatory dysfunction, and (3) polycystic ovaries on ultrasound. In 2023, it was also established that the anti-Müllerian hormone (AMH) test can be considered instead of ultrasound, with the exclusion of other aetiologies. Importantly, when irregular menstrual cycles and hyperandrogenism are present, diagnosis is simplified, and ultrasound or AMH tests are not required. In adolescents, both hyperandrogenism and ovulatory dysfunction are required, with ultrasound and AMH not recommended due to poor specificity [3].

PCOS is a complex and chronic condition in which the symptoms differ from patient to patient. Among the most frequent are the alterations in the reproductive system, such as hyperandrogenism, polycystic ovaries, abnormalities of the menstrual cycle, problems maintaining pregnancy, and infertility. In addition to these characteristics, women with PCOS often present with metabolic, clinical, and cosmetic changes that are detrimental for their quality of life. Examples of these are cutaneous alterations (acne, hirsutism, and alopecia), eye damage, overweight, and obesity [4]. Furthermore, mental health disorders such as anxiety and depression are also common in this population [5,6]. There is no definitive treatment for PCOS due to the heterogeneity of its clinical manifestations. Current treatment is multidisciplinary, as the reproductive, metabolic, cardiovascular, dermatological, and psychological characteristics of the patients must be considered. It is recommended to implement a comprehensive lifelong reproductive health plan that focuses on preconception risk factors, a healthy lifestyle, weight gain prevention, and fertility optimization. Therapeutic strategies include lifestyle changes; psychological therapy, which could be considered the first-line treatment; combined oral contraceptives; metformin or a combination of both; pharmacological agents against obesity, such as liraglutide; antiandrogenic pharmacological agents; mechanical laser and light therapies for hair reduction; and bariatric/metabolic surgery in selected cases [3]. Therefore, healthcare professionals should consider all of the above characteristics, together with each patient’s medical history, in order to develop a personalized treatment tailored to their needs [7,8].

An important issue for a deeper understanding of PCOS is the identification of the etiological factors that trigger it. It is now widely accepted that it involves strong genetic and epigenetic components. The latter is probably the consequence of alterations in the intrauterine environment during foetal life. Such early perturbations may predispose women to develop the syndrome later in life and, in the case of males, to develop endocrine and/or metabolic disorders [9]. The physiopathological components to explain the origin of PCOS include (1) a metabolic disorder resulting from insulin resistance, (2) disruptions in follicular development and steroidogenesis, (3) hyperactivity of the sympathetic innervation of the ovaries, and (4) neuroendocrine dysfunction produced by an increase in the secretion of the gonadotropin-releasing hormone (GnRH) and the luteinizing hormone (LH) [10]. In the present review, we focus on the last component by analysing the participation of the hypothalamic nuclei that regulate the activity of GnRH neurons and their link to the development of PCOS.

## 2. Methodology

We conducted bibliographical research in PubMed, Medline, and Scopus in order to identify scientific articles, published between 2010 and 2025, with evidence about the possible involvement of hypothalamic nuclei in the onset and persistence of PCOS. We also included studies describing the clinical, endocrine, and metabolic aspects of the pathology. For the search, we focused on the following combinations of keywords: “hypothalamic nuclei and PCOS”, “arcuate nucleus and PCOS”, “rostral periventricular area of the third ventricle and PCOS”, “kisspeptin and PCOS”, “neuropeptide Y and PCOS”, “endocrine aspects and PCOS”, “metabolic aspects and PCOS”, and “clinical aspects and PCOS”. We selected original research articles and relevant reviews, prioritizing those published in peer-reviewed journals, with emphasis on the most recent publications. We did not make exclusions based on the species used in the studies; therefore, both human and animal research were considered.

## 3. Clinical Features of PCOS

For the diagnosis of PCOS, it is necessary to consider different clinical features such as hyperandrogenism, oligo-anovulation, and polycystic ovarian morphology (PCOM). In addition, increases in body mass index, obesity, and metabolic dysfunction–associated steatotic liver disease (MASLD) are common characteristics that occur in women with PCOS but are not part of the diagnosis. Some of these are described below.

### 3.1. Hyperandrogenism

Since the 1990s, hyperandrogenism has been considered a central diagnostic feature of PCOS, which was recently ratified in the 2023 edition of the International Evidence-based Guidelines for the Assessment and Management of PCOS [3]. According to this document, hyperandrogenism can be evaluated either biochemically or clinically. In biochemical evaluations, free testosterone levels are assessed. Free testosterone can be estimated by the calculated free androgen index. For the detection of hyperandrogenism in PCOS, the assessment of biochemical hyperandrogenism is of great value in patients with minimal or no clinical signs of hyperandrogenism (i.e., hirsutism).

Clinical hyperandrogenism, mainly diagnosed by the presence of hirsutism, should be considered predictive of biochemical hyperandrogenism and PCOS in adult women. In addition, a comprehensive history and physical examination should be completed for symptoms and signs of clinical hyperandrogenism, including acne, female pattern of hair loss, and hirsutism. In adolescents, severe acne and hirsutism must be specially considered. It is also recommended that healthcare providers take into account the psychosocial impact that often accompanies the clinical features of hyperandrogenism [3].

### 3.2. Polycystic Ovarian Morphology

The evaluation of ovarian morphology has been part of the diagnostic criteria of PCOS since its earliest reports, in which the ovaries were described as “enlarged ovaries with an engrossed capsule” [11]. Following the Rotterdam diagnostic consensus, PCOM was defined as the presence of 12 or more follicles measuring 2–9 mm in diameter in one or both ovaries, or ovaries with a volume ≥ 10 cm^3^. Although this definition has been widely accepted by the scientific and medical community, a subsequent study established a two-fold increase in the number of follicles in the ovaries (>25) to reduce diagnosis errors [12]. The 2023 International Evidence-Based Guidelines for the Assessment and Management of PCOS suggest that the number of follicles is the most reliable ultrasound marker for diagnosing PCOM in adult women. In this case, a threshold of ≥ 20 follicles in at least one of the ovaries is indicative of the syndrome. Unfortunately, it is also stated that there are no definitive criteria to define PCOM through ultrasound in teenagers [3].

Recently, Di Michele et al. [13] conducted a study comparing various tools for the assessment of PCOS, including conventional ultrasound, 2D and 3D imaging, Doppler techniques, and artificial intelligence, with the aim of improving diagnostic accuracy. The authors report that 2D ultrasound remains a valuable and accessible tool since it allows the determination of the number of follicles per ovary, despite the limitations of probe frequency and the inherent characteristics of the patient, such as age and body mass index. For higher precision, it is recommended to use 3D ultrasound and Doppler techniques, as they allow the automatization of the follicular and stromal measurements. Regarding artificial intelligence, the authors concluded that it is a promising area of development since it could eliminate human bias in image interpretation.

### 3.3. Increases in Body Mass Index (BMI)

Overweight and obesity are common characteristics in women with PCOS [14]. However, there is wide variability in the prevalence of these factors depending on the geographical region and the population studied. Overweight is defined as a BMI between 25 and 30 kg/m^2^, while obesity is defined as a BMI greater than 30 kg/m^2^ [15]. Despite this, the exact prevalence of overweight and obesity in women with PCOS is still not known with certainty. It is important to note that the percentages of obesity in women with PCOS vary considerably depending on the country or continent where the study is conducted. For example, a meta-analysis estimated a pooled prevalence of obesity of 61% in women with PCOS [14]. The proportion of women with PCOS who are overweight, without being obese, ranges from 10% in Italy to 37% in Kuwait [16]. On the other hand, the highest rates of obesity in this population of women have been reported in studies conducted in the United States and Australia, with a prevalence of 61% and 76%, respectively [16]. Obesity is a metabolic condition characterized by an increased body mass index. However, Ezeh et al. [17] pointed out that such an index alone is not enough to assess metabolic risk, as it does not allow one to distinguish whether the weight gain is due to an increase in fat or lean mass. This, in turn, could account for the fact that there are PCOS-diagnosed women that display insulin resistance while others do not.

### 3.4. Metabolic Dysfunction–Associated Steatotic Liver Disease (MASLD)

MASLD, defined as the presence of steatotic liver disease (SLD) and one or more cardiometabolic risk factors, has emerged as the most common chronic liver condition, affecting more than one-third of the world’s adult population [18]. It encompasses a wide range of hepatic disturbances, from asymptomatic hepatic steatosis, associated with hepatocyte lesion, inflammation, and fibrosis, with or without an increase in aminotransferase levels, to hepatic cirrhosis with or without fibrosis [19]. Patients with MASLD also display obesity, insulin resistance, and type 2 diabetes mellitus. Moreover, recent evidence suggests that brain ageing is also associated with MASLD, possibly linked to low-grade systemic inflammation [20,21,22]. Several studies have shown that adults with fat accumulation in the liver have an elevated mortality rate [23]. Approximately half of the women diagnosed with PCOS also present with MASLD, which increases the risk of developing metabolic disease [24]. A meta-analysis conducted by Rocha et al. [25] found that, in general, women with both pathologies exhibit higher testosterone levels than those with PCOS alone. The authors suggest that the increase in androgen levels may contribute to the accumulation of fat in the liver.

## 4. Metabolic Alterations in PCOS

In patients with PCOS who are overweight or obese, insulin resistance is a frequently associated condition; however, several studies have shown that this is also observed in lean patients [26]. Insulin resistance is characterized by impaired glucose metabolism, which is mainly related to a reduction in insulin-mediated glucose uptake and dysregulation of lipolysis [27]. This is due to defects in the binding of insulin to its receptor or to changes in signal transduction. In this condition, cells require higher levels of insulin to respond appropriately, increasing the secretion of the hormone by the β-cells in the pancreas, leading to hyperinsulinemia despite normal glycemia. Over time, β-cell function declines, and patients may develop glucose intolerance or type II diabetes mellitus [28].

The compensatory hyperinsulinemia observed in PCOS patients provokes an overstimulation of tissues and organs, such as the ovaries, that do not normally depend on insulin for their function. Insulin and LH act synergistically on theca cells of the ovarian follicles, stimulating androgen synthesis and leading to hyperandrogenism, the main diagnostic feature of the syndrome [26]. In vitro studies have shown that insulin promotes the proliferation of theca cells and the expression of the receptor for the insulin-like growth factor 1 (IGF-1). IGF-1 is a growth factor with endocrine activity that is synthesized in the liver and other organs, including the ovary, in which it acts as an autocrine/paracrine regulator of steroidogenesis. Moreover, since the ovarian steroidogenic enzymes are similar to those in the adrenal cortex, insulin can enhance the steroidogenic response of the *zona reticularis* to adrenocorticotropic hormone (ACTH), thereby increasing androgen secretion [29].

At the molecular level, insulin binds to its receptor in oocytes, granulosa, and theca cells, stimulating the expression of genes that regulate the progression of meiosis in the former [30]. It has also been demonstrated that insulin receptors are expressed in the hypothalamus and pituitary, which explains the stimulant effect that insulin has on the secretion of follicle-stimulating hormone (FSH) and LH [30,31]. Considering the number of patients with PCOS who develop insulin resistance, it is believed that hyperinsulinemia contributes to hyperandrogenism through direct stimulation of ovarian steroidogenesis. Women with PCOS have reduced levels of sex hormone-binding globulin (SHBG). This glycoprotein, produced in the liver, binds to most sex steroids. Elevated plasma insulin levels are responsible for the high concentration of androgens in PCOS. Insulin inhibits hepatic secretion of SHBG and increases the availability of androgens [31,32]. The importance of SHBG in PCOS lies in the fact that measurements of serum SHBG concentrations can act as an appropriate predictor of response to pharmacological treatment in infertile women with PCOS [32].

Several studies have shown an increase in the IGF-1/insulin-like growth factor binding protein-1 (IGFBP-1) ratio in patients with PCOS. The higher availability of IGF-1 enhances androgen synthesis by theca cells. In addition, IGF-1 stimulates oestrogen synthesis by granulosa cells, as IGF-1 acts in conjunction with FSH and LH to modulate aromatase expression. Like insulin, IGF-1 indirectly regulates ovarian steroidogenesis by acting on the components of the hypothalamic–pituitary axis. There is evidence to suggest that IGF-1 induces the expression of the gene encoding the GnRH precursor and also stimulates the release of gonadotropins from the anterior pituitary. In women with PCOS, the administration of metformin, a drug that increases insulin sensitivity, increases IGFBP-1 levels, reducing the IGF-1/IGFBP-1 ratio and thereby decreasing IGF-1 availability in peripheral tissues. Consequently, the synthesis of 17-hydroxyprogesterone, testosterone, and androstenedione is reduced in these patients [33].

## 5. Endocrine Imbalance in PCOS

Women with PCOS display a neuroendocrine disorder that involves the hypothalamic–pituitary–ovarian axis and affects the regulation of several steroid and protein hormones. Among these hormonal changes, GnRH secretion is increased, which in turn stimulates LH secretion, while FSH levels remain normal or even decrease, resulting in an elevated LH/FSH ratio [34,35,36]. This hormonal imbalance leads to hyperandrogenism, as LH acts on theca cells of ovarian follicles, stimulating the activity of the enzymes 17α-hydroxylase and 17, 20-lyase, which participate in the biosynthesis of androgens and from progestogens [37,38].

Elevated levels of AMH, produced by granulosa cells, have also been reported in patients with PCOS, and this has been associated with changes in androgen levels [39], suggesting that AMH may contribute to the development of hyperandrogenism in PCOS [40]. The AMH type II receptor (AMHR2) is expressed in theca cells, indicating that AMH is active in this cell type. However, in vitro studies show that AMH inhibits androgen production in human theca cells [40]. The alteration in androgen secretion, combined with decreased serum FSH levels, impairs ovarian follicle development, leading to atresia and anovulation [41,42,43].

As a result of peripheral conversion of androgens into oestrogens, mainly in the adipose tissue, an increase in circulating estrone and oestradiol levels has been described in women with PCOS [42,44,45]. This hyperestrogenism has also been attributed to increased aromatase activity in granulosa cells [41]. Oestrogens have mitogenic effects, and their elevated levels in these women may increase the risk of developing cancers in the endometrium, breast, and ovary [37,41,42,44]. However, there is ongoing debate regarding whether oestrogen levels are actually elevated in women with PCOS. Clinical and experimental studies suggest that oestrogen levels in these patients are typically comparable to those of healthy women [46], possibly due to the reduced number of granulosa cells in ovarian cysts [47] and decreased FSH secretion [48].

Based on the diagnostic criteria discussed earlier, four PCOS phenotypes have been identified:

1. Phenotype A (classic type I PCOS): characterized by hyperandrogenism, oligo/anovulation, and PCOM.
2. Phenotype B (classic type II PCOS): with hyperandrogenism and oligo/anovulation, but without PCOM.
3. Phenotype C (ovulatory PCOS): with hyperandrogenism and PCOM, but with preserved ovulatory cycles.
4. Phenotype D (normoandrogenic PCOS): with chronic anovulation and PCOM, but without clinical or biochemical hyperandrogenism [38,41,49,50].

Some research groups have reported that these phenotypes share common endocrine and clinical characteristics, while others are specific to individual phenotypes [49].

In addition to high levels of testosterone and dehydroepiandrosterone sulphate (DHEAS), the first three phenotypes are also characterized by the presence of hirsutism, significantly lower SHBG levels, and a high body mass index. In phenotypes A and B, an elevated LH/FSH ratio is also reported, which is more pronounced in phenotype A due to an exacerbated release of LH, suggesting that it represents a more severe form of PCOS [49]. In phenotype D, an increase in LH levels that leads to a higher LH/FSH ratio has also been reported; however, total and free testosterone levels are lower [49]. All four phenotypes show similar oestradiol levels. Phenotype B shows normal ovarian volume, whereas all other phenotypes are characterized by increased ovarian volume. Based on these observations, some authors have proposed that phenotype A is the more common and severe type of the syndrome, in which LH may be directly related with the PCOM, particularly when compared with phenotype B. On the other hand, phenotype C appears to be the mildest form, as patients exhibit testosterone levels and body mass index values that are intermediate between those of phenotype A and healthy women [49].

Recent studies have shown that the prevalence of the four phenotypes of PCOS varies with age. In a population of 596 women with the syndrome, phenotype A was identified as the most common, with a prevalence of 61.4% among young women approximately 20 years of age. However, this percentage declined to 2.9% in women aged 50. A similar pattern was observed for phenotype B (from 16.3% to 4.4%) and phenotype D (from 20.5% to 7.6%). In contrast, the prevalence of phenotype C increased with age, from 0.7% to 25.5%. These changes in phenotype distribution were accompanied by endocrine abnormalities, with serum levels of LH, testosterone, dehydroepiandrosterone, and AMH decreasing, while oestradiol increased [51].

## 6. The Neuroendocrine Regulation of the Reproduction

Ovarian function is regulated by the hypothalamic–pituitary–ovarian (HPO) axis. In mammals, the GnRH neurons originate during embryonic development in the olfactory placode and the neural crest. They eventually migrate to regions of the forebrain and hypothalamus [52]. However, the consolidation and final localization of these neurons in the brain varies among species. In rodents, GnRH neurons are distributed along the medial septum, the vertical limb of Broca’s diagonal band, the vascular organ of the lamina terminalis, the medial preoptic area, and the anterior hypothalamic area [53]. In humans, these neurons are mainly concentrated in the basal medial hypothalamus and the arcuate nucleus (also known as the infundibular nucleus) [54]. In other species, such as sheep, GnRH neurons are primarily located in the vascular organ of the lamina terminalis and, to a lesser extent, in the anterior preoptic area and the basal medial hypothalamus [55]. Hypothalamic GnRH neurons integrate signals from different regions of the nervous system and release the neuropeptide at the median eminence. GnRH is then transported via the capillaries of the hypothalamic–pituitary portal system to the anterior pituitary, where it binds to membrane receptors on gonadotrophs, stimulating the synthesis and release of FSH and LH. Both gonadotropins reach the ovaries through the systemic circulation and bind to their receptors on ovarian follicular cells, regulating follicular development and steroidogenesis [56].

GnRH neurons are regulated both directly and indirectly through a wide range of chemically diverse neurotransmitters, including kisspeptin, gamma-aminobutyric acid (GABA), vasoactive intestinal peptide (VIP), glutamate, noradrenaline, RFamide-related peptide 3 (RFRP-3), neuropeptide Y (NPY), arginine vasopressin (AVP), serotonin, dopamine, acetylcholine (ACh), and AMH, among others [53,57,58]. For example, AMH participates in the regulation of GnRH neuron activity in mice and humans via its AMHR2 receptor. In adult female mice and humans, AMHR2 is expressed by >50% of the GnRH neurons located in the preoptic region (POA), including the organum vasculosum of the lamina terminalis, as well as in more rostral areas such as the septum and the diagonal band of Broca [57]. In the same study, the activity of GnRH neurons was analysed in brain sections exposed to different concentrations of AMH within the physiological range. GnRH neurons from animals in proestrus or diestrus that were treated with 4 nM AMH showed an increase in excitation from 0.93 ± 0.55 to 2.47 ± 0.24 Hz, with a prolonged activity duration of 13.2 ± 1.2 min. These results show that AMH exerts a powerful stimulating influence on GnRH neurons. Tissue explants containing the ME were also obtained and stimulated with AMH for 4 h. Treatment resulted in a fourfold increase in GnRH release. In addition, to examine the effects of AMH on gonadotropin secretion in vivo, AMH was microinjected at a concentration of 3 mM directly into the lateral ventricle of female mice in diestrus. It was observed that AMH induced an increase in LH release 15 min after microinjection. These findings support a central role for AMH in stimulating GnRH neuron activity in rodents [57].

## 7. The Neuroendocrine Regulation of the PCOS

It has been proposed that the onset and persistence of PCOS may result from alterations in hypothalamic and extrahypothalamic neuronal circuits involved in the regulation of GnRH secretion, particularly those involved in the feedback mechanisms by which ovarian hormones modulate it [59]. From a neuroendocrine perspective, dysfunctions in GnRH pulsatility are considered a key pathophysiological component of PCOS. Specifically, an increase in the pulsatile release of GnRH is known to preferentially stimulate the release of LH over FSH, resulting in an elevated LH/FSH ratio of approximately 2:1. In response to LH hyperstimulation, theca cells synthesize and release excessive amounts of androgens, while oestrogen secretion is reduced, thereby eliminating the negative feedback on the hypothalamus [35]. Taken together, these alterations could explain the hyperandrogenism characteristic of women with PCOS [60].

### 7.1. The Involvement of the Arcuate Nucleus (ARC) and the Rostral Periventricular Area of the Third Ventricle (RP3V) in PCOS

**Figure 1:**
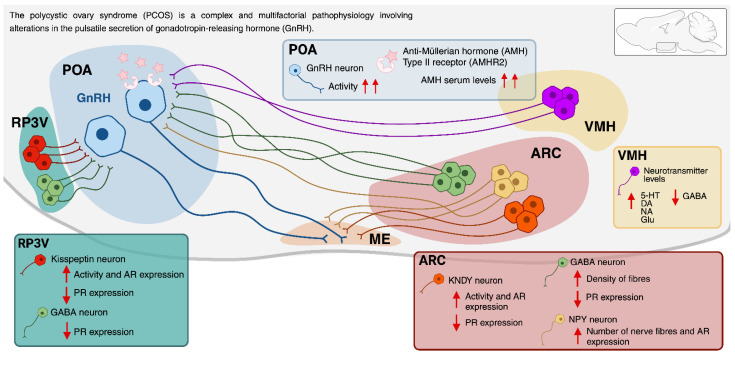
The schematic illustrates the hypothalamic nuclei, neurotransmitters, neuropeptides, and steroid hormone receptors involved in the regulation of gonadotropin-releasing hormone (GnRH) pulsatility and secretion in murine models of polycystic ovary syndrome (PCOS). Abbreviations: preoptic area (POA), arcuate nucleus (ARC), rostral periventricular area of the third ventricle (RP3V), ventromedial nucleus of the hypothalamus (VMH), median eminence (ME), gamma aminobutyric acid (GABA), neuropeptide Y (NPY), anti-Müllerian hormone (AMH), progesterone receptors (PR), androgen receptors (AR), serotonin (5-HT), dopamine (DA), noradrenaline (NA), and glutamate (Glu). Created in Biorender. Roberto Calderón Ramos. (2025). https://app.biorender.com/profile/template/details/t-68a3619fa57f620bd0610e03-regulacion-nerviosa-de-la-neurona-gnrh-en-el-sopqreview-2025/?source=profile&username=laboratorio_reproducci%C3%B3n.

In the search for the origin of PCOS, several studies have examined hypothalamic nuclei and areas that may contribute to the development and maintenance of the syndrome, among them the ARC and RP3V [61]. Both regions contain neurons that express oestrogen receptors and kisspeptin, the most potent stimulator of GnRH release known to date. In this context, the ARC and RP3V act as intermediaries between ovarian oestrogenic signals and GnRH neurons, mediating both the negative and positive feedback effects of oestrogens. In addition, GABA, NPY, and kisspeptin synthesized by ARC neurons may be key players in the mechanisms underlying the changes in GnRH secretion observed in PCOS (Figure 1) [62,63,64].

### 7.2. Expression of Steroid Hormone Receptors in the ARC and RP3V and Its Relationship with PCOS

The kisspeptin neurons in the ARC and RP3V express progesterone receptors (PR), androgen receptors (AR), and oestrogen receptors (ER), which modulate their responsiveness to gonadal steroids and, consequently, their role in the regulation of GnRH secretion [65]. Throughout most of the ovarian cycle, steroid hormones suppress GnRH neuronal activity via negative feedback mechanisms [66]. It has been hypothesized that the neuroendocrine abnormalities associated with PCOS result from hyperstimulation of GnRH neurons by kisspeptin neurons in the ARC and RP3V, driven by the activation of their androgen receptors [67].

Moore and Campbell [68] reported that, in a murine model of PCOS induced by prenatal androgen exposure, there was a 44% and 58.3% decrease in the PR immunoreactive cells in the RP3V and the ARC, respectively. No differences were found in the number of ER-immunoreactive cells in either region. However, a significant increase in AR-immunoreactive cells was observed in the RP3V (Figure 1). These results clearly indicate that the expression of progesterone and androgen receptors is altered in rodents with PCOS [69]. Years later, the same group demonstrated that specifically in the kisspeptin neurons of the ARC, AR expression increases while PR expression decreases (Figure 1). This effect is accompanied by reduced synaptic activity from local GABAergic and glutamatergic neurons, which also show reduced PR expression. Based on these findings, it was postulated that in the PCOS model, testosterone may act directly on kisspeptin neurons or on steroid-sensitive presynaptic populations, ultimately impairing steroid feedback [67].

### 7.3. The Role of Kisspeptin in PCOS

As previously mentioned, kisspeptin is a neuropeptide critically involved in the regulation of GnRH neuronal secretory activity [69]. In rodents, the main populations of kisspeptin neurons are located in the ARC and RP3V, whereas in humans they are primarily found in the infundibular nucleus and, to a lesser extent, in the POA [70]. These neurons co-express the alpha isoform of the oestrogen receptor (ERα), which is pivotal for mediating the feedback actions of oestradiol. They act as interneurons, relaying information about changes in oestradiol levels through the reproductive cycle to GnRH neurons, which do not express ERα. The population of kisspeptin neurons located in the ARC co-expresses NKB and Dyn and is referred to as KNDy neurons. These cells are active when levels of oestradiol are basal, stimulating the secretion of GnRH in low-frequency pulses and thereby mediating the negative feedback of oestrogens [70]. In contrast, RP3V kisspeptin neurons become active when oestradiol levels rise prior to ovulation, increasing the frequency of GnRH secretion and thereby triggering the preovulatory LH surge [71].

### 7.4. Alterations in the Kisspeptin System in Murine Models of PCOS

It has been proposed that polymorphisms or mutations in the *kiss1* gene, which encodes the precursor of kisspeptin, may disrupt the HPO axis [72], resulting in abnormal GnRH secretion, an altered LH/FSH ratio, increased androgen synthesis, and anovulation [73]. Esparza et al. [64] described abnormalities in the kisspeptin system of a mouse model of PCOS induced by letrozole, an aromatase inhibitor. These animals exhibited the classic diagnostic features of PCOS, which are associated with a significant increase in the frequency and amplitude of LH pulses. At the hypothalamic level, in situ hybridization revealed increased kiss1 expression in the ARC. This population of kisspeptin neurons also showed increased activity compared to those in the control group. These findings indicate that, in the letrozole-induced PCOS model, altered LH pulsatility may be driven by abnormal activity of ARC kisspeptin neurons.

In the letrozole model of PCOS, selective inactivation of ARC kisspeptin neurons using designer receptors exclusively activated by designer drugs (DREADDs) leads to a reduction in LH pulse frequency. This intervention restores serum LH and testosterone levels to values similar to those observed in control animals. Moreover, a reduction in ovarian expression of the *Cyp17a1*, *Fshr*, and *Amh* genes was also observed. Based on these findings, the authors propose that increased kisspeptin input to GnRH neurons is a key factor in the exacerbated secretion of GnRH and LH observed in this PCOS model [74].

### 7.5. Evidence of Alterations in the Kisspeptin System in Women with PCOS

Umayal et al. [75] evaluated blood levels of kisspeptin and testosterone in a cohort of 55 adolescent females. They found significantly elevated levels of both hormones in participants with PCOS compared to healthy controls (kisspeptin: 4873 nmol/L vs. 4127 nmol/L; testosterone: 4713 nmol/L vs. 3415 nmol/L; *p* ≤ 0.05). A significant association between elevated kisspeptin levels and the presence of PCOS was also observed. Based on these results, the authors suggest that kisspeptin could serve as a potential biomarker for the early diagnosis of PCOS.

A pilot study involving 12 women aged 18–42 diagnosed with non-ovulatory PCOS evaluated the ovulatory response to twice-daily administration of kisspeptin over a 21-day period. The treatment increased serum LH and oestradiol levels; however, only two women had follicular development with subsequent ovulation, one had a dominant follicle but did not ovulate, and, in the remaining participants, neither follicular development nor ovulation occurred [76]. These results indicate that kisspeptin can stimulate the secretion of GnRH and gonadotropins, albeit with low efficacy. This supports the need for personalized treatments that consider the characteristics and PCOS phenotype of each patient [77].

In KNDy neurons, NKB stimulates the release of kisspeptin and is therefore considered a potential therapeutic target in PCOS. Administration of an NKB receptor antagonist (AZD4901) in women with PCOS lowers the frequency of LH pulses and the serum levels of LH and testosterone [78]. In a separate study, twice-daily treatment with AZD4901 for 28 days in women aged 19–31 with PCOS also reduced LH pulsatility when compared to healthy controls. Interestingly, administration of kisspeptin alone had no effect on LH pulses, but when administered after AZD4901 treatment, it reduced pulsatility to values similar to those of the control group. These results indicate that both NKB and kisspeptin are involved in the dysregulated GnRH secretion observed in women with PCOS. The authors propose that a complex interaction between these two neuropeptides is required to regulate the precise pattern of GnRH secretion, as well as the amount of neuropeptide released [79].

A novel therapeutic strategy for PCOS involves the administration of inositol derivatives, such as myoinositol. In a study involving 14 women with PCOS, serum levels of kisspeptin, FSH, LH, oestradiol, and testosterone were measured during the menstrual phase, both before and after a 3-month treatment with 750 mg of myoinositol administered daily. Following treatment, kisspeptin and oestradiol levels decreased, while gonadotropin and testosterone levels remained unchanged. A reduction in ovarian size was also reported. Based on these results, the authors suggest that myoinositol may have beneficial effects in PCOS and could serve as a potential starting point to refine current therapeutic strategies [80]. Together, the studies discussed above highlight the relevance of kisspeptin in the development and persistence of PCOS and support the need to further explore this line of research.

### 7.6. The Role of Other ARC Neurotransmitter Systems in PCOS

*Gamma-aminobutyric acid.* GABA is another neurotransmitter involved in the regulation of GnRH secretion. Moore and colleagues [81] showed that, in mice with PCOS, the density of GABAergic fibres originating in the ARC and projecting to GnRH neurons increased (Figure 1). In addition, the somas of these GABAergic neurons exhibit reduced expression of PR (Figure 1), which may contribute to progesterone desensitization in PCOS mice. Disruption of progesterone-mediated feedback at the level of the ARC could enhance GABAergic neurotransmission to GnRH neurons. Although GABA is generally considered an inhibitory neurotransmitter, there is evidence suggesting that it exerts an excitatory effect on GnRH neurons, particularly in prenatally androgenized rodents, via GABA_A_ receptors [82,83]. Based on these findings, Moore and colleagues proposed a model in which GABA influences reproductive function by increasing the frequency of GnRH and gonadotropin secretion, which could lead to the other alterations observed in women with PCOS [81].

To date, the role of androgens in the onset of PCOS has not been clearly established. Considering that GABAergic neurons express AR, it has been hypothesized that androgens may act on these neurons to disrupt GnRH secretion. Using Cre-Lox technology, a research group generated knockout mice lacking AR expression specifically in GABAergic neurons. These animals were exposed to high levels of androgens during the prenatal or peripubertal stages but showed no increase in the GABAergic input to GnRH neurons. Based on these results, it was proposed that foetal or peripubertal programming that leads to the development of PCOS depends on androgen action on GABAergic neurons located in the ARC [62].

*Neuropeptide Y.* NPY is another neuropeptide synthesized in the ARC that participates in the regulation of GnRH secretion [84]. Terasawa mentioned that NPY pulses are necessary for the pulsatile release of GnRH [85]. In a murine model of PCOS induced by prenatal androgen exposure, it was also reported that NPY neurons in the ARC project their axons to the rostral POA and the anterior hypothalamic area (AHA), where they make contact with GnRH cell bodies. In the same study, it was found that up to one-third of GABAergic neurons in the ARC co-express NPY. This subpopulation of GABA/NPY neurons does not express PR or ERα, but does express AR. These findings led the authors to suggest that GABA/NPY neurons are not the primary targets of prenatal androgens and therefore do not undergo anatomical remodelling. This implies that the changes in the GABA-GnRH system described in PCOS occur within a yet unidentified subpopulation of GABAergic neurons. In addition, they suggest that NPY neurons in the ARC may exhibit increased sensitivity to androgens [86].

Coutinho et al. [63] demonstrated that selective activation of ARC neurons co-expressing NPY and agouti-related peptide (AgRP) results in a reduction in the frequency of GnRH/LH pulses in both male and female mice, including animals subjected to prenatal androgenization to induce a PCOS-like phenotype. Moreover, in these animals, optogenetic activation of NPY/AgRP fibres near GnRH cell bodies in the POA also reduced LH pulsatility. Based on this, the authors propose that activation of NPY/AgRP neurons, which are primarily involved in the circuit that regulates energy homeostasis and hunger, may cause infertility under conditions of negative energy balance. This mechanism, however, could also represent a potential therapeutic strategy to reduce LH pulsatility in pathologies such as PCOS.

In addition to modulating reproductive processes, NPY is one of the key substances responsible for regulating appetite and controlling eating behaviour. Thus, in patients with PCOS, NPY not only modulates fertility by regulating the release of GnRH/LH but also plays an important role in maintaining energy balance, body weight, and circulating glucose and lipid levels [84]. In the 1990s, Baranowska’s group observed a relationship between NPY levels and body weight, reporting significantly elevated plasma NPY levels in both obese and non-obese patients with PCOS. In addition, Guzelkas’ group [87] conducted a cross-sectional study, reporting that serum NPY and leptin concentrations are higher in adolescent girls aged 14 to 18 years diagnosed with PCOS, with or without obesity. Based on these findings, the authors proposed that NPY contributes to the development of the syndrome in women, regardless of obesity. The authors suggested that elevated NPY levels in both obese and non-obese patients with PCOS indicate a role in the pathogenesis of the condition, independent of obesity. Although there is an increasing number of studies implicating NPY in the regulation of the reproductive and metabolic functions of PCOS [84], further research is needed into the role of this neurotransmitter, which modulates key aspects such as reproduction and metabolism.

### 7.7. Role for Anti-Müllerian Hormone in the Regulation of GnRH and Gonadotrophins in PCOS

A study using a murine model of PCOS induced by prenatal androgen exposure (PNA) analysed whether AMH concentrations were increased and whether changes in AMH levels correlated with LH and FSH levels. Unlike women with the syndrome, plasma AMH concentrations did not differ between the control and treatment groups (Ctrl: 19.83 ngmL^−1^ ± 3.37; PNA: 19.9 ngmL^−1^ ± 3.33). However, a significant positive correlation was observed between AMH and LH levels in PNA-treated mice (Pearson’s R = 0.71; P = 0.01). Plasma FSH concentration did not differ between the two groups (Ctrl: 0.37 ngmL^−1^ ± 0.01; PNA: 0.36 ngmL^−1^ ± 0.06). Furthermore, no correlation was observed between AMH and FSH levels. These observations thus suggest that AMH levels increase before the rise in LH in women at risk of developing PCOS. This study establishes a new role for AMH as a central regulator of the HPO axis under both physiological and pathological conditions (Figure 1) and raises the intriguing hypothesis that the perturbation of the AMH-dependent regulation of GnRH release could play a critical role in the development of PCOS [57].

Among the neurological implications observed in women with PCOS, several studies have shown that they report greater sexual dysfunction and distress compared to those without the condition [88]. Clinical studies also report an increased risk of low arousal, desire, and sexual satisfaction [89]. Silva et al. (2022) investigated the possible specific neural pathways that modulate sexual behaviour in patients with PCOS. To this end, they investigated in a prenatal AMH-treated mice (PAMH), a model that induces the characteristics of PCOS, whether prenatal excess AMH modulates the sexual circuits that promote sexual dysfunction in female mice with PCOS. Among the nuclei that modulate sexual circuits is the VMH, which is considered the centre of specialized neurons that modulate sexual behaviour [90]. This nucleus contains neurons that express neuronal nitric oxide synthase (nNOS), the enzyme responsible for the production of nitric oxide, which stimulates female sexual behaviour. The authors reported decreased expression of nNOS and progesterone receptor (PR) in the VMH. These anatomical changes were also associated with a significant deterioration in sexual receptivity in female mice with PCOS. With these results, the researchers proposed a brain pathway that could explain the aetiology of low sexual desire in PCOS while pointing to possible therapeutic approaches to restore normal sexual function in these women [91].

### 7.8. The Role of VMH in PCOS

VMH is a brain structure that, in addition to regulating sexual behaviour, also regulates energy metabolism, and it has been suggested that it plays a role in the regulation of reproductive functions. Kim et al. [92] analysed the effect of the absence of steroidogenic factor 1 (SF-1), a factor synthesized exclusively by VMH neurons in the brain, using a mouse model with a central nervous system-specific deletion of SF-1 (SF-1 KOnCre;F/−). In these animals, a decrease in fertility was observed, which was associated with a reduced ovulatory response, alterations in the oestrous cycle, and changes in the secretion of gonadotropins and steroid hormones.

In animal models of PCOS induced by neonatal exposure to a single dose of oestradiol or testosterone, it has been observed that the concentration of hypothalamic neurotransmitters is altered, which may be associated with the manifestation of PCOS in adulthood. A study by Sotomayor-Zárate [93] used a neonatal rat model exposed to oestradiol valerate (EV) or testosterone propionate (TP) during the first 12 h of life. In adulthood, the concentration of various neurotransmitters was assessed in the ARC and VMH, along with ovarian morphology. It was found that exposure to EV increased the concentrations of serotonin, dopamine, noradrenaline, and glutamate, while decreasing the concentrations of GABA (Figure 1). In contrast, TP exposure increased glutamate and decreased serotonin levels in the same region. Regarding ovarian morphology, the number of primordial follicles per ovary was reduced by 30% in EV-treated rats and by 60% in rats exposed to TP. Furthermore, neonatal treatment with EV or TP produced a significant increase in cystic follicles and an absence of corpus luteum [94]. The authors suggest that the early exposure to EV or TP has differential effects on ovarian follicular development and on VMH-ARC neurotransmitters, consistent with the early activation of hypothalamus-mediated control of reproduction in adult animals neonatally exposed to EV or TP, where oestradiol permanently increases glutamatergic and noradrenergic neurotransmission. It is therefore suggested that the administration of EV before the maturation of the hypothalamus produced a permanent excitatory effect that altered its functioning into adulthood [93].

As mentioned above, PCOS alters metabolic and reproductive physiology and psychological processes in affected women. Given that PCOS is known to be associated with hyperandrogenism and occurs in both lean and obese women, Ressler’s group [95] used a DHT-induced PCOS animal model to evaluate whether metabolic and behavioural alterations are influenced by dietary exposure. Additionally, they assessed c-Fos expression to identify the brain regions potentially involved in mediating these differences. To this end, some of the DHT-treated rats were assigned to a high-fat obesogenic diet (HFD) to represent an obese PCOS model or to a nutrient-adapted low-fat diet (LFD) to model lean PCOS. The authors reported that hyperandrogenism in this animal model drives overall body weight gain, glucose intolerance, anxiety behaviours, and stress responsiveness. However, palatable HFD consumption exacerbates adiposity, insulin resistance, and depressive behaviours. Therefore, in the treatment and characterization of human PCOS, stratification by metabolic profile and appropriate dietary intervention may be beneficial in controlling the constellation of reproductive, metabolic, and psychological aspects of PCOS. At the hypothalamic level, c-Fos-positive cells were counted in the preoptic nucleus (PON), suprachiasmatic nucleus (SCN), PVN, VMH, and ARC. In the PVN, increased c-Fos expression was observed in animals fed HFD. Regardless of the diet received (HFD or LFD), animals treated with DHT showed reduced c-Fos expression in the VMH and ARC. Overall, these findings suggest that hyperandrogenism is associated with a generally dampened stress response [95].

Alterations in the hypothalamus can lead to human diseases, such as obesity, diabetes mellitus and insipidus, hypertension, and amenorrhea [94]. Furthermore, recent studies suggest that changes in the hypothalamus also lead to neurodegenerative diseases, such as amyotrophic lateral sclerosis, Huntington’s disease, and Alzheimer’s disease [96]. Specifically, in animal models with PCOS, the VMH appears to be one of the hypothalamic nuclei that modifies its neuronal activity due to changes in the synthesis of different neurotransmitters that possibly modulate the activity of GnRH neurons in the POA. In addition, the VMH is involved in several metabolic processes associated with PCOS comorbidities, as well as psychological alterations related to sexual function.

## 8. Recent Therapeutic Strategies Employed in the Treatment of PCOS

Considering the multifactorial origin of PCOS, therapeutic strategies should be tailored to the clinical characteristics and priorities of each patient, with a focus on improving metabolic, reproductive, and psychological symptoms [3]. According to the International Evidence-Based Guidelines for the Assessment and Treatment of PCOS (2023), first-line treatment includes lifestyle interventions, especially for women who are overweight or obese. These interventions consist of a healthy diet, regular physical activity, and behavioural support. These changes have been shown to promote weight loss, improve insulin sensitivity, reduce androgen levels, and support ovarian function, while also contributing to an improved quality of life [97,98].

In the reproductive context, letrozole, an aromatase inhibitor, is recommended as a first-line pharmacological treatment for ovulation induction in women with PCOS who wish to conceive. Women with PCOS who receive this treatment show a higher pregnancy rate (29.0% vs. 15.4%), follicular development (77.2% vs. 52.7%), and a higher live birth rate (25.4% vs. 10.9%) compared to those treated with clomiphene citrate [99]. The latter, widely used in the past, is now considered a second-line alternative in cases of letrozole unavailability or contraindication. Metformin, which improves insulin sensitivity and can reduce androgen levels, is also considered a complementary option, especially in patients with intolerance to other drugs or with marked metabolic characteristics [100].

Some additional strategies have been proposed, although they are not considered as first-line treatments. These include specific nutritional interventions such as the Mediterranean diet, which has been associated with anti-inflammatory effects and metabolic benefits [101]. The use of supplements such as myo-inositol and D-chiro inositol has also been explored, as they may improve insulin sensitivity and ovarian function in some cases; however, current evidence is insufficient to support widespread recommendation [3]. Similarly, omega-3 supplementation in women with PCOS and obesity has been shown to improve pregnancy rates by 29.6% and reduce body mass index [102]. On the other hand, Hu et al. [103] showed that in women with PCOS and severe obesity, bariatric surgery is significantly more effective than pharmacological therapy, as it consistently promotes weight loss after surgery, restores menstrual cycles, and improves both hormonal and metabolic profiles.

Despite lifestyle changes, few patients with PCOS who are overweight or obese manage to significantly reduce their body weight. For this reason, drugs such as liraglutide, exenatide, and semaglutide, which are glucagon-like peptide-1 (GLP-1) agonists, have been used to improve reproductive outcomes by optimizing the metabolic profile in these women [104]. However, it has been suggested that these drugs could also directly modulate the reproductive axis. GLP-1 is an anorexigenic gastrointestinal hormone principally produced by L cells in the small intestine and colon [105].

In murine models, intracerebroventricular (ICV) injection of GLP-1 has been shown to increase serum LH concentrations and promote progesterone and oestradiol secretion. Furthermore, expression of the mRNA encoding its receptor (GLP-1R) has been characterized in the hypothalamus, pituitary gland, and ovary [106]. Using an in vitro model, GLP-1 has been shown to increase the firing rate of GnRH neurons [107]. Similarly, it has been reported that GnRH neurons are innervated by GLP-1-producing fibres and that their activity increases when GLP-1-secreting neurons are optogenetically stimulated [108]. In sheep, administration of GLP-1 or exendin-4, a GLP-1 agonist, to the median eminence or intravenously, respectively, stimulates LH secretion [109]. Taken together, these findings suggest that GLP-1 agonists could directly normalize GnRH pulse secretion in women with PCOS.

Although GnRH neurons express GLP-1R, KNDy neurons in the ARC also express this receptor [110], which adds to the complexity of the neural network that could be modulated by GLP-1 agonists in women with PCOS. GLP-1 has been shown to stimulate kiss1 expression in hypothalamic cell lines (rHypoE-8), an effect that is inhibited by the exendin 9–39 fragment, a GLP-1R antagonist [111]. Likewise, it has been shown that these neurons form appositions with GLP-1-secreting nerve fibres and that their firing rate increases when the GLP-1R is stimulated [110]. Given that the activity of GnRH neurons is influenced by kisspeptin, it is possible that, through this pathway, GLP-1 agonists could support improved reproductive function in women with PCOS. In summary, PCOS treatment should be personalized and based on up-to-date evidence, prioritizing safe and effective interventions according to each patient’s needs.

## 9. Acupuncture as an Alternative Therapy for the Treatment of PCOS

Acupuncture, a fundamental component of Chinese medicine, is a technique used to maintain good health and treat various diseases [112]. Electroacupuncture is a new form of acupuncture treatment that capitalizes on electrical stimulation [113]. Both techniques have been and are used as alternative therapies to treat PCOS and also to investigate the neuroendocrine mechanisms responsible for the development and persistence of PCOS [114,115]. Previous research has demonstrated that EA can effectively restore the oestrous cycle, improve the ovarian polycystic morphology, regulate circulating sex hormone levels, improve insulin resistance, and increase ovarian AR expression in PCOS rats [114]. Some research conducted on rodents has attempted to elucidate the role of this technique in the regulation of GnRH neurons.

Xu et al. [114] showed that stimulation at the so-called “Guan Yuan point” (CV 4) in a rat model of letrozole-induced PCOS restored the progression of the oestrous cycle and improved the PCOM, accompanied by a reduction in LH and testosterone serum levels. In addition, acupuncture treatment decreased GnRH levels and downregulated the expression of kiss1 and the kisspeptin receptor (GPR54) in the ARC, both of which were elevated before stimulation. These results suggest that acupuncture may attenuate the hyperactivity of mechanisms responsible for GnRH secretion, likely via the inhibition of the kisspeptin system. The same study reported a reduction in the expression of ERα and an increase in AR expression in KNDy neurons, suggesting an alteration in the sensitivity to steroid hormones in these neurons, which may explain their aberrant pattern of activity in PCOS. In this context, acupuncture could be a suitable option to reduce androgen synthesis and its effects in the brain.

There is evidence suggesting that electroacupuncture can restore steroid hormone levels in PCOS models to values comparable to those of the control animals. This effect appears to depend on mechanisms involving both the inhibition of GnRH secretion and the regulation of the expression of AR in the hypothalamus. In a murine model of PCOS, Li et al. [115] reported an increased expression of NPY in the ARC. Electroacupuncture stimulation at SP6, ST36, and CV6 points resulted in decreased expression of the neuropeptide, absence of ovarian cysts, and restoration of oestrous cyclicity.

The NPY receptor Y2 is known to participate in the regulation of reproductive functions since its activation results in the suppression of GnRH/gonadotropin secretion [116]. The expression of this receptor in the hypothalamus of rats with PCOS is lower than in controls, which is reversed following the stimulation of the aforementioned points. The authors concluded that electroacupuncture signals reach the brain and activate NPY and Y2-expressing neurons, which in turn results in a decrease in GnRH pulses in PCOS animals, attenuating the symptoms [115].

Electroacupuncture applied to women with PCOS has been effective for improving hyperandrogenism and insulin resistance [113,117], increasing ovulation frequency, and improving follicular growth [118,119]. However, research is still being conducted to try to elucidate the possible mechanisms that lead to the beneficial effects of electroacupuncture on the reproductive or metabolic implications caused by the syndrome. In this regard, Li’s research group [120] studied the signalling pathways of insulin receptor/phosphatidylinositol 3-kinase/glucose transporter 4 (IRS-1/PI3K/GLUT4) substrates, as this is an important pathway in insulin metabolism, and any alteration in one of these pathways can cause insulin resistance. Currently, it has been reported that in PCOS patients with insulin resistance, GLUT4 expression is decreased, and electroacupuncture can improve oocyte quality [120]. However, the mechanism by which electroacupuncture improves oocyte quality was not described at that time. A study by Xiang et al. [119] reported that electroacupuncture improves oocyte quality in PCOS patients by increasing the expression of IRS-1 and PI3K genes and upregulating GLUT4 expression in the cell membrane. This enhances glucose uptake and utilization in peripheral tissues and reduces insulin resistance through the IRS-1/PI3K/GLUT4 signalling pathway. Furthermore, it can improve oocyte quality and the proportion of high-quality embryos, thereby improving IVF outcomes in patients with PCOS-related infertility.

Electroacupuncture on the cheeks is a technique that involves a system of micro-needles and produces rapid analgesic effects [121]. This technique has been applied to women with PCOS and infertility problems caused by the syndrome [122]. In two clinical cases of women aged 23 and 32 diagnosed with PCOS, treatment was administered every other day, with each session lasting 30 min for a period of 2 to 3 months. At the end of the treatment, both women successfully ovulated and became pregnant. Throughout the acupuncture treatment, follicular development was observed, leading to ovulation, conception, and childbirth in both patients. This, to some extent, suggests that acupuncture on the cheeks has the potential to promote follicular development and induce ovulation. The authors propose that the mechanism by which cheek acupuncture promotes ovulation involves precise regulation of the HPO axis and reduction of testosterone levels through holographic modelling and precise modulation of the sacral region, which influences the hypothalamus and the parasympathetic nervous system. Cheek acupuncture therapy stands out for its standardized approach and painless procedure, making it suitable for clinical use and widespread dissemination [122].

In June 2025, Wang’s group [123] systematically evaluated the clinical efficacy of acupuncture and metformin on insulin resistance in women with PCOS through a meta-analysis study. The researchers showed that electroacupuncture and abdominal acupuncture significantly reduced insulin resistance compared to metformin, as measured by fasting glucose and insulin levels assessed by the homeostasis model of insulin resistance (HOMA-IR) [124]. When comparing the two techniques (electroacupuncture and abdominal acupuncture) in terms of their effectiveness in improving HOMA-IR, electroacupuncture was identified as the most effective intervention for reducing HOMA-IR in women with PCOS-related insulin resistance, followed by abdominal acupuncture.

The evidence presented above suggests that various forms of acupuncture may serve as therapeutic tools to alleviate PCOS symptoms in women. In addition, the technique should be personalized, as the frequency of sessions and voltage will depend on whether or not the woman is obese.

## 10. Conclusions

PCOS is a complex disorder with a multifactorial aetiology, involving endocrine and metabolic alterations that may result from anatomical and physiological abnormalities in the central nervous system, particularly in hypothalamic nuclei such as the ARC, RP3V, and VMH. These nuclei play a key role in the regulation of the HPO axis, and alterations in their activity appear to contribute to increased GnRH and gonadotropin pulsatility. This, in turn, leads to hyperandrogenism and alterations in ovarian morphology and function. In addition, insulin resistance and altered metabolic signalling at the hypothalamus suggest a complex interaction between hypothalamic nuclei in the onset and maintenance of PCOS. The evidence discussed in this article highlights the need for new therapeutic strategies that focus on alterations in the communication between different neural populations in the ARC and the GnRH neurons, taking into account the PCOS phenotype of each patient. Based on this, it is pivotal to continue the research of the neural mechanisms implicated in PCOS.

## Data Availability

Data are contained within the article.
